# Comparing mortality between coronary artery bypass grafting and percutaneous coronary intervention with drug-eluting stents in elderly with diabetes and multivessel coronary disease

**DOI:** 10.1007/s00380-015-0746-1

**Published:** 2015-09-28

**Authors:** Ryo Naito, Katsumi Miyauchi, Hirokazu Konishi, Shuta Tsuboi, Manabu Ogita, Tomotaka Dohi, Kan Kajimoto, Takatoshi Kasai, Hiroshi Tamura, Shinya Okazaki, Kikuo Isoda, Taira Yamamoto, Atsushi Amano, Hiroyuki Daida

**Affiliations:** 1 Department of Cardiovascular Medicine, Juntendo University School of Medicine, Tokyo, Japan; 2 Department of Cardiovascular Surgery, Juntendo University School of Medicine, Tokyo, Japan

**Keywords:** Percutaneous coronary intervention, Coronary artery bypass graft, Elderly, Diabetes mellitus, Multivessel disease

## Abstract

Coronary artery disease is a critical issue that requires physicians to consider appropriate treatment strategies, especially for elderly people who tend to have several comorbidities, including diabetes mellitus (DM) and multivessel disease (MVD). Several studies have been conducted comparing clinical outcomes between percutaneous coronary intervention (PCI) and coronary artery bypass graft (CABG) in patients with DM and MVD. However, elderly people were excluded in those clinical studies. Therefore, there are no comparisons of clinical outcomes between CABG and PCI in elderly patients with DM and MVD. We compared all-cause mortality between PCI with drug-eluting stents (DES) and CABG in elderly patients with DM and MVD. A total of 483 (PCI; *n* = 256, CABG; *n* = 227) patients were analyzed. The median follow-up period was 1356 days (interquartile range of 810–1884). The all-cause mortality rate was not significantly different between CABG and PCI with DES groups. The CABG group had more patients with complex coronary lesions such as three-vessel disease or a left main trunk lesion. Older age, hemodialysis, and reduced LVEF were associated with increased long-term all-cause mortality in a multivariable Cox regression analysis. The rate of all-cause mortality was not significantly different between the PCI and CABG groups in elderly patients with DM and MVD in a single-center study.

## Introduction

Industrialized countries are experiencing aging societies. Japan has one of the highest life expectancies in the world with life expectancies of 79.6 and 86.3 years in Japanese men and women, respectively [1]. An aging society is associated with increased morbidity from atherosclerotic diseases. Coronary artery disease (CAD) has been a critical issue that requires physicians to consider appropriate treatment strategies for elderly people who tend to have several comorbidities, including diabetes mellitus (DM), hypertension, and multivessel coronary disease (MVD) [2–5]. In the past few decades, several studies have been conducted comparing clinical outcomes between percutaneous coronary intervention (PCI) and coronary artery bypass graft (CABG) in patients with DM and MVD [6–8]. A recent large-scale randomized study demonstrated superior clinical outcomes in patients with DM and MVD who underwent CABG versus PCI with drug-eluting stents (DES) [9]. However, elderly people were excluded in those clinical studies. Therefore, there have been no comparisons of clinical outcomes between CABG and PCI with DES in elderly patients with DM and MVD. The aim of this study was to compare long-term clinical outcomes between CABG and PCI with DES in elderly patients with DM and MVD.

## Methods

### Study population

We analyzed data from consecutive patients in our database aged 65 and older with DM and MVD who underwent a PCI with DES or a CABG at Juntendo University Hospital (Tokyo, Japan) between April 2004 and December 2008. A revascularization strategy for each patient was determined based on the patient’s background, such as age, active daily living, and comorbid diseases, in our heart team conference with cardiovascular physicians and surgeons.

### Clinical outcomes

The clinical outcome was all-cause mortality. Information regarding outcomes was collected during clinical visits, via telephone interviews with the patients or from their referring physicians. Our institutional review board approved the protocol of this study, which was implemented in accordance with the principles established in the Declaration of Helsinki and our institutional ethics policy.

### Definitions

Based on the American Heart Association Classification, we defined MVD as CAD when two or three vessels were visually assessed with more than 75 % stenosis. ACS was defined as unstable angina pectoris (UAP), non-ST segment elevation myocardial infarction (NSTEMI), or STEMI. UAP was defined as angina at rest or in an accelerating pattern with negative cardiac biomarkers, with or without ECG changes indicative of myocardial ischemia (for example, ST segment depression or transient elevation or new T-wave inversion). Myocardial infarction was defined as an increase (≤2-fold) in serum creatinine kinase and troponin T positivity. DM was defined as glycated hemoglobin A1c (HbA1c) with an NGSP value ≥6.5 % or under treatment with anti-diabetic agents or insulin. We converted HbA1c [Japan Diabetes Society (JDS)] values to HbA1c (NGSP) units using the following equation: NGSP (%) = 1.02 × JDS (%) + 0.25 % [10]. Hypertension was defined as a systolic blood pressure ≥140 mmHg and a diastolic blood pressure ≥90 mmHg or under treatment with anti-hypertensive medications. Dyslipidemia was defined as triglyceride levels ≥150 mg/dL, low-density lipoprotein cholesterol (LDL-C) levels ≥140 mg/dL, high-density lipoprotein cholesterol (HDL-C) levels <40 mg/dL, or under medication dyslipidemia. We defined current smokers as individuals who smoked at the time of admission or who had quit within 1 year before the study period. Renal dysfunction was defined as an estimated glomerular filtration rate (eGFR) of <60 mL min^−1^ 1.73 m^2^ calculated using the modification of diet in renal disease (MDRD) equation which was modified with a Japanese coefficient using baseline serum creatinine [11].

### Statistical analyses

Continuous variables were expressed as mean values with standard deviations (SD). Categorical data were expressed as counts and percentages. Comparisons of continuous variables were performed with an unpaired *t* test or Mann–Whitney *U* test. Categorical variables were analyzed by Chi-squared tests or Fisher’s exact probability test. Unadjusted cumulative event rates were estimated by Kaplan–Meier methods and compared using a log-rank test between the groups. To identify predictors of outcomes, a univariable Cox regression analysis was performed including age, gender, BMI, hypertension, dyslipidemia, current smoking, prior MI, LDL-C, HDL-C, TG, hemoglobin A1c, hemodialysis, left ventricular ejection fraction, eGFR, and CABG as independent variables. Hazard ratios (HR) and 95 % confidence intervals were also calculated. Variables with *p* < 0.1 in the analysis were analyzed by multivariable Cox regression analysis. A *p* value < 0.05 was considered to be statistically significant. All the data were analyzed using JMP version 10.0 for Windows (SAS Institute, Cary, NC, USA).

## Results

A total of 483 (PCI; *n* = 256, CABG; *n* = 227) patients were analyzed in this study. The median follow-up period was 1356 days (interquartile range of 810 to 1884). The baseline characteristics are shown in Table 1. The mean age was similar between the two groups (72.7 ± 5.3 and 72.7 ± 5.3 in the PCI and CABG groups, respectively). The percentage of male gender was higher in the PCI group. The patients in the CABG group were more likely to have a prior myocardial infarction, reduced LV function, and triple-vessel disease or left main trunk lesion, while the PCI group included more patients undergoing hemodialysis. The lipid profiles were similar between the groups. The glycated hemoglobin value was higher in the CABG group. The medications for secondary prevention of coronary artery disease, such as DAPT, statin, ACE-I/ARB, and β-blocker, were administered in more patients in the PCI group than the CABG group. The percentage of left internal thoracic artery (LITA) use was 98.2 %. The rates of bilateral internal thoracic artery (BITA) use and total artery revascularization (TAR) were 55.9 and 26.4 %, respectively (Table 1).

**Table 1:** Baseline characteristics

	PCI group, (*N* = 256)	CABG group, (*N* = 227)	*p* value
Age	72.7 ± 5.3	72.7 ± 5.1	0.9
Male, *n* (%)	200 (78.1)	155 (68.3)	0.017
BMI, kg/m^2^	24.0 ± 3.3	23.8 ± 3.4	0.7
Hypertension, *n* (%)	197 (77.0)	168 (74.0)	0.45
Dyslipidemia, *n* (%)	196 (76.6)	156 (68.7)	0.053
Smoking, *n* (%)	180 (58.6)	142 (62.6)	0.2
Family history, *n* (%)	71 (27.7)	40 (17.6)	0.008
Hemodialysis, *n* (%)	19 (7.4)	3 (1.3)	0.0013
Prior MI, *n* (%)	76 (29.8)	101 (44.5)	0.0019
Total cholesterol, mg/dL	182.8 ± 31.7	179.0 ± 39.3	0.2
LDL-C, mg/dL	111.2 ± 26.4	106.4 ± 27.9	0.055
HDL-C, mg/dL	44.9 ± 13.1	46.0 ± 13.0	0.36
Triglyceride, mg/dL	134.0 ± 73.5	133.3 ± 66.7	0.9
HbA1c (NGSP), %	7.0 ± 1.1	7.3 ± 1.1	0.015
LVEF, %	61.9 ± 11.5	56.7 ± 12.9	<0.001
eGFR, mL/min/1.73 m^2^	64.1 ± 25.0	60.3 ± 27.3	0.1
Medication, (%)			
Aspirin	97.7	93.8	0.08
Dual antiplatelet therapy	97.7	15.3	<0.0001
Statin	69.8	23.7	<0.0001
ACE-I/ARB	51.4	28.7	<0.0001
β-blocker	51.4	33.2	<0.0001
Diseased vessel, (%)			
Triple-vessel disease, (%)	53.5	74.0	<0.0001
Left main trunk, (%)	19.6	37.4	0.0002
Left internal thoracic artery, (%)	NA	223 (98.2)	NA
Bilateral internal thoracic artery, (%)	NA	127 (55.9)	NA
Total arterial revascularization, (%)	NA	60 (26.4)	NA
Type of coronary artery disease			0.0005
Stable angina pectoris	88.3	76.2	
Acute coronary syndrome	11.7	23.8	

*NA* not applicable, *BMI* body mass index, *MI* myocardial infarction, *LDL-C* low-density lipoprotein cholesterol, *HDL-C* high-density lipoprotein cholesterol, *HbA1c* glycated hemoglobin, *LVEF* left ventricular ejection fraction, *eGFR* estimated glomerular filtration rate, *ACE-I* angiotensin-converting enzyme inhibitor, *ARB* angiotensin receptor blocker

### Clinical outcomes

**Fig. 1:**
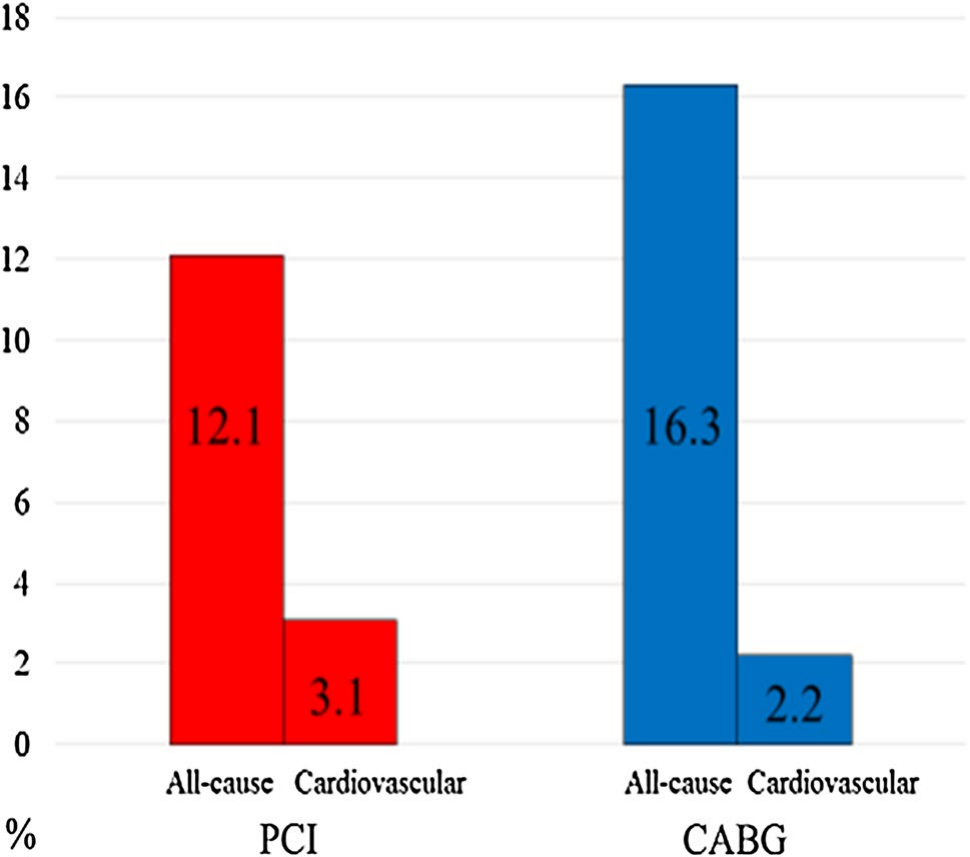
All-cause mortality rate was 12.1 and 16.3 % in PCI- and CABG-treated groups, respectively. Cardiovascular mortality rate was 3.1 and 2.2 % in PCI- and CABG-treated groups, respectively

**Fig. 2:**
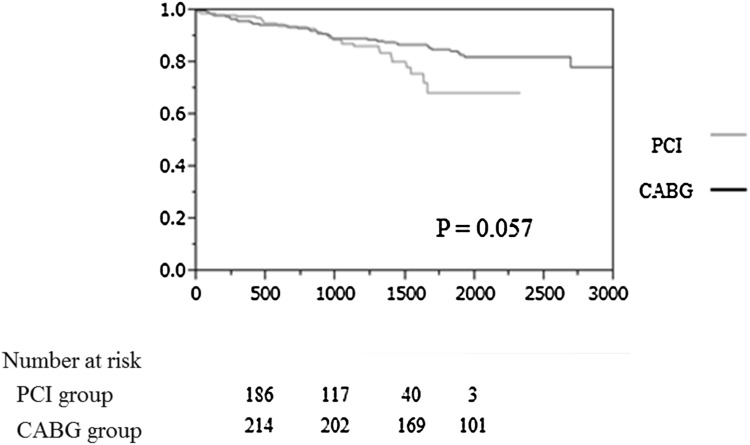
Cumulative event-free survival curves for all-cause mortality. Kaplan–Meier curves for all-cause mortality show no significant difference between PCI- and CABG-treated groups, respectively

All-cause mortality was observed in 31 patients (12.1 %) in the PCI group and 37 (16.3 %) in the CABG group (Fig. 1). The cardiovascular mortality rates were 3.1 and 2.2 % in the PCI and CABG groups, respectively. The event-free curves for all-cause mortality were in favor of the CABG group, although the difference was not statistically significant (Fig. 2). The univariable Cox regression analysis for all-cause mortality showed that older age, hemodialysis, and reduced LVEF were associated with an increase in the long-term all-cause mortality. The CABG was not associated with the clinical outcome in the univariable Cox regression analysis. No association was observed between the use of LITA, BITA, or TAR and the clinical outcome in the analysis. After adjusting for confounding variables, age, hemodialysis, and LVEF remained significant (Table 2).

**Table 2:** Cox regression analysis for all-cause mortality

	Univariable			Multivariable		
	HR	95 % CI	*p* value	HR	95 % CI	*p* value
Age, year	1.1	1.07–1.16	<0.0001	1.1	1.06–1.16	<0.0001
Male gender	0.78	0.48–1.31	0.34			
BMI, per 1 kg/m^2^ increase	0.93	1.01–1.07	0.08	0.95	0.87–1.02	0.17
Hypertension, yes	1.78	0.99–3.48	0.056	1.61	0.83–3.46	0.17
Prior MI, yes	1.50	0.92–2.42	0.21			
HbA1c, per 1 % increase	0.88	1.10–1.14	0.26			
HD, yes	4.55	1.98–9.15	0.001	3.51	1.27–8.30	0.018
LVEF, per 1 % increase	0.98	0.96–0.99	0.01	0.97	0.95–0.99	0.0031
CABG, yes	0.61	0.36–1.02	0.06	0.73	0.40–1.38	0.3

*BMI* body mass index, *MI* myocardial infarction, *HbA1c* glycated hemoglobin, *HD* hemodialysis, *LVEF* left ventricular ejection fraction, *CABG* coronary artery bypass graft

## Discussion

The main results of the study were as follows: (1) The all-cause mortality rate was not significantly different between the CABG and PCI with DES in the patients ≥65 years old, DM, and MVD. (2) The patients who underwent CABG had more complex coronary lesions, such as three-vessel disease or left main trunk lesion. (3) Older age, hemodialysis, and reduced LVEF were associated with an increase in long-term all-cause mortality in the multivariable Cox regression analysis.

A meta-analysis comparing PCI and CABG in patients with DM and MVD demonstrated that there was no significant difference in mortality or MI between the two procedures [12]. In contrast, a large-scale randomized study with 1900 patients with DM and MVD showed that mortality and MI rates were better with the CABG procedure than PCI [9]. However, studies comparing PCI and CABG often exclude elderly patients, who are considered to have more comorbidities and higher mortality rates associated with revascularization therapy [2, 13, 14]. Thus, these results do not reflect clinical practices in an aging society. Although several studies have investigated the comparative effectiveness between PCI and CABG in elderly patients [15, 16], the study populations were not diabetic patients without MVD, but with left main coronary artery disease. Our study is novel because it focused on elderly patients with DM and MVD.

In this study, the all-cause mortality rate was not different between the CABG and PCI with DES groups, despite the higher-risk profiles of the patients in the CABG group in terms of prior myocardial infarctions, reduced LV function, and triple-vessel disease or left main trunk lesion. A possible explanation is that CABG not only is a revascularization therapy for focal coronary artery lesions but may also have a beneficial effect on a wide range of myocardial perfusion because of increased blood flow through the grafts. Thus, CABG might lead to favorable clinical outcomes by improving cardiac function, although we had no data regarding improvement in cardiac function, such as LVEF, following the CABGs. The other reason for the lack of significant differences in all-cause mortality was that the decision of revascularization strategy for each patient that was determined in our heart team conference by cardiovascular physicians and surgeons was appropriate. Because CABG is highly invasive in contrast to PCI, selecting a revascularization therapy depends not only on complexity of the lesion but also on a patient’s medical history and comorbidities. In previous clinical trials, higher-risk surgical patients, such as the elderly and those with more comorbid diseases, were excluded. Therefore, selecting a revascularization therapy for a patient with CAD and DM requires a more thorough discussion of the patient’s coronary anatomical features and lesion characteristics, age, comorbid conditions, cardio-pulmonary function, and frailty. The thorough discussion among the cardiovascular physicians and surgeons to decide a revascularization strategy for each patient could affect the prognosis following each revascularization, although the effect of these heart team discussions on clinical outcomes was not assessed in this study.

In the multivariable Cox regression analysis, older age, hemodialysis, and low LVEF were associated with all-cause mortality. A previous report that has demonstrated the prognostic impact of reduced LVEF on clinical outcomes in patients with coronary artery disease [17] supports our findings. Hemodialysis has also been demonstrated to be associated with worse clinical outcomes following coronary revascularization [18–20].

### Limitations

First, heterogeneity between the PCI-treated group and CABG-treated group had a potential effect on the results of this study, although the Cox regression analysis was performed to minimize the effect of the different patients’ background between two groups. In addition, undetermined factors might have affected the results because our data were derived from a longitudinal cohort. Second, other endpoints, including target vessel revascularization and cerebral infarction, were not assessed in the present study because all the study population was not followed by our institution after discharge. The outcome data of the patients were limited to mortality by their referring doctors who performed the follow-up, which was a major limitation considering that almost all clinical trials comparing PCI and CABG assessed the events as primary or secondary endpoints. Third, although the revascularization strategy was determined considering the patient’s background and comorbid diseases in the conference with cardiovascular physicians and surgeons, a quantitative assessment of coronary artery lesion complexity or patient background, such as SYNTAX2 or EuroScore, was not performed. Finally, this was a retrospective study in a single institution with a relatively small number of study population, and therefore this study was underpowered for the clinical outcome and the results may not be directly generalizable.

## Conclusions

The rate of all-cause mortality was not significantly different between PCI and CABG in elderly patients with DM and MVD in this single-center study. Age, HD, and LVEF were significantly associated with long-term clinical outcomes in this population.
